# Differences in the influenza-specific CD4 T cell immunodominance hierarchy and functional potential between children and young adults

**DOI:** 10.1038/s41598-018-37167-5

**Published:** 2019-01-28

**Authors:** Ian Shannon, Chantelle L. White, Amy Murphy, Xing Qiu, John J. Treanor, Jennifer L. Nayak

**Affiliations:** 1 Department of Pediatrics, Division of Infectious Diseases, University of Rochester Medical Center, 601 Elmwood Ave, Box 690, Rochester, NY 14642 USA; 2 Department of Biostatistics and Computational Biology, University of Rochester Medical Center, 265 Crittenden Blvd, Box 630, Rochester, NY 14642 USA; 3 Biomedical Advanced Research and Development Authority (BARDA)/HHS/ASPR, Influenza and Emerging Diseases Division 21J14, 200 C St SW, Washington, DC 20515 USA

## Abstract

Studies of the B cell repertoire suggest that early childhood influenza infections profoundly shape later reactivity by creating an “imprint” that impacts subsequent vaccine responses and may provide lasting protection against influenza strains within the same viral group. However, there is little known about how these early childhood influenza exposures shape CD4 T cell reactivity later in life. To investigate the effect of age on influenza-specific CD4 T cell specificity and functionality, reactivity in cohorts of 2 year old children and young adult subjects was compared. Intracellular cytokine staining was used to determine the viral antigen specificity and expression levels of various cytokines following stimulation of peripheral blood mononuclear cells with complete peptide pools representing the entire translated sequences of the pH1, H3, HA-B, NP, and M1 proteins. We found that the influenza protein-specific immunodominance pattern in children differs from that in young adults, with much lower reactivity to the NP internal virion protein in young children. Alterations in CD4 T cell functionality were also noted, as responding CD4 T cells from children produced less IFNγ and were less likely to express multiple cytokines. These differences in the repertoire of influenza-specific CD4 T cells available for recall on influenza challenge in early childhood could possibly contribute to early imprinting of influenza-specific immunity as well as the increased susceptibility of children to this viral infection.

## Introduction

Influenza is a respiratory virus that accounts for substantial morbidity, mortality, and a high economic burden due to the occurrence of yearly seasonal epidemics and unpredictable pandemics, with the highest disease burden in the young pediatric and elderly populations^1–4^. Although inactivated influenza vaccines (IIV) have been available since the 1940s and are currently in widespread use^5^, IIVs are weakly immunogenic and are susceptible to antigenic mismatch as a result of frequent antigenic drift driven by immunologic pressure to escape from neutralizing antibodies^6,7^. As such, there is great interest in development of a more universally protective influenza vaccine that is able to provide broad protection against multiple viral subtypes, including potentially pandemic strains^8^.

While neutralizing antibody directed against the HA protein is the most well established correlate for immunity against influenza^9,10^, CD4 T cells have multiple functions associated with protection from infection^11–13^. These cells are essential for the provision of cognate help to B cells, enabling the formation of germinal centers and development of high affinity neutralizing and non-neutralizing antibody responses^14,15^. CD4 T cells also contribute to CD8 T cell positioning, effector function, and memory formation^16,17^, the establishment of an early innate immune response upon viral infection^18,19^, and are able to provide direct cytotoxicity^20,21^. Adults have been shown to have a broad and diverse influenza-specific CD4 T cell repertoire, with cytokine production enriched for IFNγ and typically considered to be “Th1-biased”^22^. However, there is much less known about influenza-specific CD4 T cell specificity and function in children.

Historically children have been first exposed to influenza via infection, however current recommendations are for prime-boost doses of influenza vaccine to be administered once a child is at least 6 months of age and yearly thereafter. As a result, many children now have their initial influenza exposure through administration of trivalent or quadrivalent IIV rather than via natural infection. As current IIVs are enriched for HA proteins with only limited amounts of internal virion proteins and trace, if any, innate immune activators, there is currently a great deal of debate regarding how vaccination primes CD4 T cell mediated immunity, particularly in early childhood.

To better understand how early vaccination establishes influenza-specific CD4 T cell responses, we used multiparameter flow cytometry to evaluate cytokine expression and specificity in a well characterized cohort of 2 year old children with previous IIV immunization but without a history of either live attenuated influenza vaccine administration or natural influenza infection. Influenza-specific CD4 T cell reactivity in these children was compared to young adult subjects with multiple past influenza encounters. We identified differences in both the specificity and functionality of influenza-specific CD4 T cells between these subject cohorts, with children having less elaboration of IFNγ upon antigenic stimulation and decreased immunodominance of the internal virion proteins when compared to adults. These data comparing the influenza-specific CD4 T cell repertoire in children versus adults may provide insight into how the anti-influenza CD4 T cell response evolves over time as well as potential approaches that could be used to positively influence the early childhood response to influenza vaccination.

## Materials and Methods

### Human subjects

Following approval from National Institutes of Health Division of Microbiology and Infectious Diseases as well as the University of Rochester Research Subjects Review Board (protocol 07-0043), blood was obtained from a group of 20 young adult healthy subjects 20 to 28 years of age in 2011 or 2012. Pediatric blood samples were obtained from a group of 10 two year old subjects following approval from the University of Rochester Research Subjects Review Board (protocol RSRB00058437) in fall 2015. In completion of this study, all experimentation guidelines of the United States Department of Health and Human Services and the University of Rochester were followed and study procedures were performed in accordance with the ethical standards of the Helsinki Declaration. All subjects or their parents provided written informed consent prior to study participation.

### Isolation of PBMCs from human blood

Within 6 hours of isolation, blood was gently spun and plasma was removed. PBMCs were then isolated using density gradient centrifugation and were frozen at a controlled rate in fetal bovine serum (FBS) containing 10% DMSO at a density of approximately 5 million cells per ml. Frozen PBMCs were thawed into RPMI-1640 (Gibco) containing 10% fetal calf serum and were rested overnight at 37 °C and 5% CO_2_ prior to use.

### Synthetic peptides and libraries

17-mer peptides overlapping by 11 amino acids encompassing the entire translated sequences of the viral proteins were obtained from the Biodefense and Emerging Infections Research Repository, National Institute of Allergy and Infectious Diseases, National Institutes of Health. The peptide arrays included A/California/04/09(H1N1) hemagglutinin (pH1; NR-15433), A/New York/384/2005(H3N2) hemagglutinin (H3; NR-2603), A/New York/348/2003(H1N1) nucleoprotein (NP; NR-2611) and matrix protein (M1; NR-2613), and B/Florida/04/2006 hemagglutinin (HA-B; NR-18972). A peptide pool encompassing 111 peptides from Sin Nombre Virus (NM H10) glycoprotein precursor protein (NR-4764) was used as a negative control. All peptides from a given protein were pooled, with each peptide present at a final concentration of 1 μM in assays.

### Intracellular Cytokine Staining (ICS)

Human PBMCs were thawed, rested, and stimulated with the negative, pH1, H3, HA-B, NP, or M1 peptide pools for 16 hours, with brefeldin A and monensin (BD Biosciences) added for the last 8 hours of stimulation. Cells were then washed and stained for 30 minutes with Fixable Live/Dead Aqua (ThermoFisher Scientific). Surface and intracellular staining was performed as per published protocol^23^. Antibodies used included CD8 (RPA-T8), CD4 (RPA-T4), CD3 (SK7), CD45RA (HI100), IL-2 (MQ1-17H1), IFNγ (B27), and CD69 (FN50) (BD Biosciences) as well as TNFα (MAb11) and CD19 (HIB19) (Biolegend), with cell fixation and permeabilization performed using the BD Cytofix/Cytoperm kit (BD Biosciences). Fluorochrome-stained UltraComp e-Beads (Invitrogen) or ArC Amine Reactive Compensation Beads (ThermoFisher Scientific) were used for compensation. Data was acquired using a BD LSR-II instrument, configured with 488, 633, 407, and 532-nm lasers using FACS DIVA software (BD Bioscience). Sequential gating was performed using FlowJo version 10 (Tree Star). Activated cells of interest were defined as CD69+ CD4+ T cells (CD3+, CD8−) positive for cytokine staining (IFNγ, IL-2, or TNFα). Throughout the manuscript, data are presented per 10^6^ CD4 T cells with background subtracted. Reactivity to the negative peptide pool was generally negative to extremely low when quantified by expression of IFNγ and IL-2 (average reactivity of 20 and 40 cytokine-producing cells per million CD4 T cells, respectively), with higher background production of TNFα noted (average of 96 TNFα producing cells per million CD4 T cells). Pie graphs were created using SPICE software (NIAID), while bar and scatter plot graphs were created using Prism 7 (GraphPad) and boxplots were created using R software (version 3.2.0).

### Statistical analysis

Two group comparison testing between pediatric and young adult data was performed using the Wilcoxon rank-sum test after computing summary values and performing mean imputation for missing data or outliers, with the resulting P-values adjusted by the Benjamini-Hochberg procedure to control for false discovery at a 0.05 level. This test was utilized as it is a robust nonparametric two group comparison method less sensitive to the presence of outliers. Two-way analysis of variance (ANOVA) was used to understand the associations between a continuous outcome variable (such as cytokine production by CD4 T cells) and two covariates (such as cohort and stimulation condition), followed by *post hoc* analyses with Sidak multiple comparison adjustment to test for significant differences between the pediatric and young adult data.

## Results

To evaluate for differences in influenza-specific CD4 T cell specificity and functionality between the pediatric and young adult populations, we enrolled a well-defined cohort of 10 pediatric subjects who were 2 years of age. These subjects had blood drawn per study protocol in the fall of 2015. All children had received prior influenza vaccinations with IIV but none had a documented history of acute influenza infection or had ever been vaccinated with a live attenuated influenza vaccine. This subject cohort was compared to a group of 20 young adult students between the ages of 20 and 28 enrolled under the University of Rochester Campus Influenza Study who had blood drawn per study protocol in late spring/summer of either 2011 or 2012. The majority of subjects in both cohorts had documented immunization with the seasonal influenza vaccine in the past (100% of pediatric, 90% of adult subjects), with most of the pediatric subjects and 50% of the adult subjects having documented influenza vaccine administration in the previous year (Table 1). None of the subjects had been diagnosed with a recent acute influenza infection. Cryopreserved PBMCs were thawed and rested overnight. PBMCs were then stimulated with complete pools of overlapping peptides representing the entire translated sequences of the pH1, H3, HA-B, NP, or M1 proteins for 16 hours to quantify CD4 T cell reactivity, with brefeldin A and monensin added for the last 8 hours to block cytokine secretion. Multiparameter flow cytometry was then performed to quantify CD4 T cell surface marker expression and cytokine production simultaneously on the small sample sizes available from young children.

**Table 1:** Demographic information.

	Pediatric	Young adult
Total subjects examined	10	20
Age (average yrs, range)	2.3 (2–2.83)	22 (20–28)
Sex (# male, %)	5 (50%)	11 (55%)
Documented previous vaccination (#, %)	10 (100%)	18 (90%)
Documented vaccination the previous year (#, %)	9 (90%)	10 (50%)
Documented pH1N1 vaccine ever (#, %)	10 (100%)	13 (65%)

**Figure 1:**
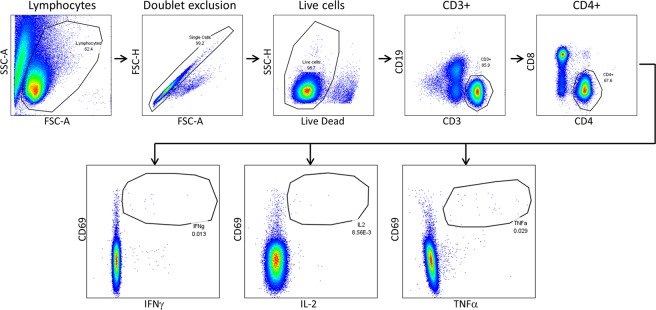
Gating schematic utilized for analysis of intracellular cytokine staining data. Using FlowJo version 10, sequential gating was applied to multiparameter flow cytometric data. Debris was first excluded, followed by gating to exclude doublets and non-viable cells. Cells were then sequentially gated on CD3+ CD19− cells, followed by CD4+ CD8− cells. Activated cytokine producing cells were gated on using CD69 combined with IFNγ, IL-2, or TNFα, with gates established based upon the staining present in positive and FMO control samples.

**Figure 2:**
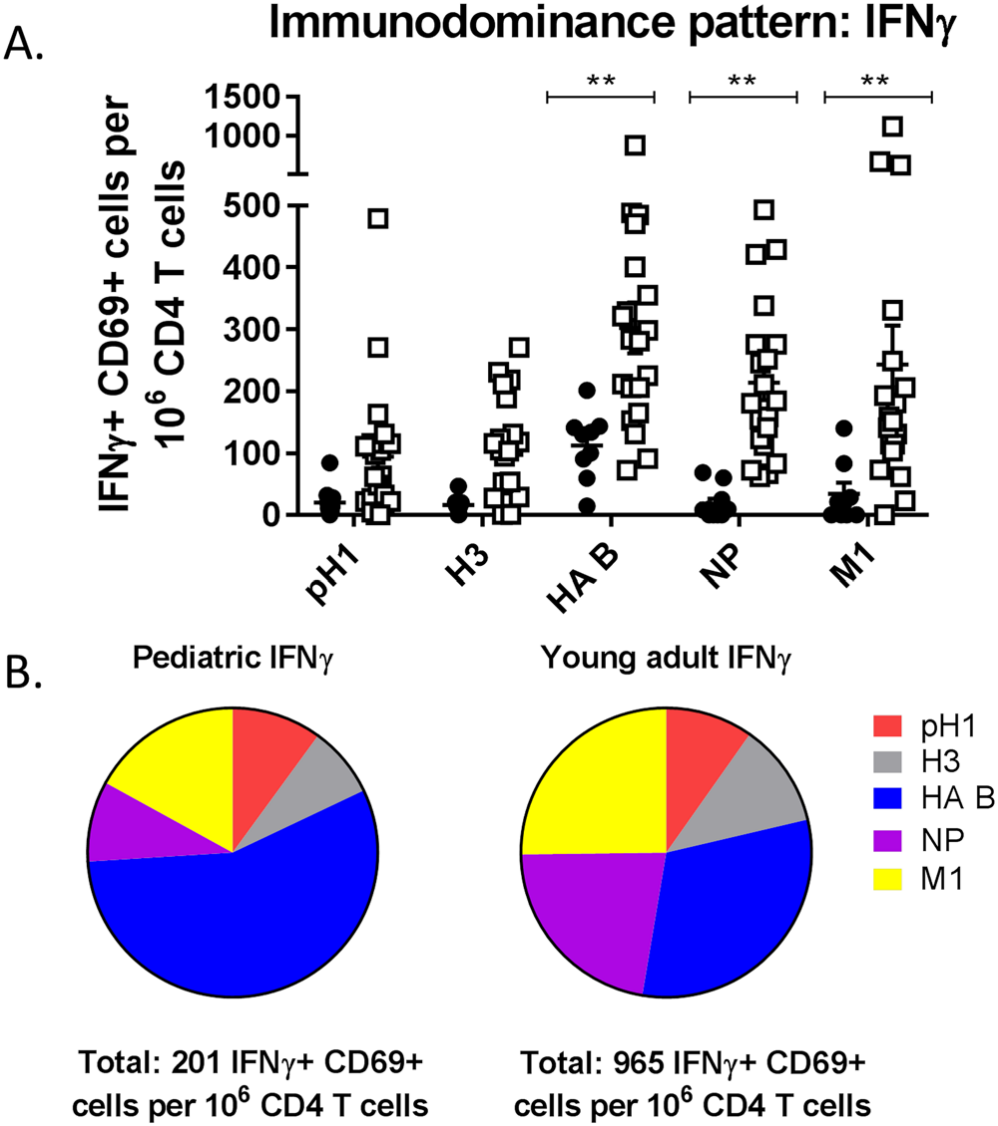
There are significant differences in influenza specific CD4 T-cell immunodomince patterns between children and young adults when quantified by INFγ production. PBMCs were stimulated with the indicated peptide pools for 16 hours, with cytokine secretion blocked with brefeldin A and monensin for the last 8 hours of incubation. Cells were then stained as described in Materials and Methods and gated using the strategy outlined in Fig. 1. In Panel A the frequency of activated IFNγ producing cells per million CD4 T-cells was quantified after subtracting background. Statistical testing was completed using a 2-way ANOVA followed by *post hoc* comparisons with Sidak multiple comparison adjustment to assess for statistical significance between children (solid circle) and young adults (open square). Panel B demonstrates the immunodomince hierarchy as a percentage of the total response. Each colored slice represents a stimulation condition as indicated in the Figure, with total summed CD4 T cell reactivity presented below the respective pie graphs.

Following exclusion of debris and doublets, cells were gated on live, CD3+, CD4+ cells to evaluate CD4 T cell specificity and function, with cytokine expression measured by gating on activated (CD69+) cytokine positive cells (Fig. 1). As IFNγ is considered a classical Th1 cytokine and influenza-specific CD4 T cell responses are typically Th1 biased, we initially evaluated the immunodominance hierarchy by quantifying IFNγ-producing CD4 T cells, with overall immunodominance by protein calculated as the frequency of IFNγ+ CD69+ cells per million CD4 T cells in Fig. 2A and as a fraction of the total response in Fig. 2B. Young adults had greater total influenza-specific CD4 T cell reactivity when quantified by IFNγ production, with about 4.8-fold more total IFNγ+ CD69+ CD4 T cells when compared to children when all specificities were summed (total of 965 IFNγ+ CD69+ cells per million CD4 T cells in adults versus 201 in children). In addition to the overall lower frequency of IFNγ-producing CD4 T cells in children, there were several other notable differences between these cohorts. When activated, IFNγ+ cells were quantified as a fraction of the total response, there was very little reactivity to the NP protein detectable in the cohort of 2 year old children. This contrasted with what was seen in the adult population, where the CD4 T cell response to NP was immunodominant. Unlike with NP, the HA-B-specific CD4 T cell response appeared to be established at a very early age, with greater than expected reactivity to HA-B even prior to children having a history of an influenza B infection. This implies that early IIV immunizations establish a high frequency of HA-B-specific CD4 T cells, with this pattern reinforced further upon subsequent influenza exposures throughout life. In contrast, reactivity to NP and M1 was quite low, suggesting that the quantity of internal virion proteins and/or the amount of inflammatory signaling within IIV does not appear to prime CD4 T cell reactivity to these proteins in previously uninfected children.

**Figure 3:**
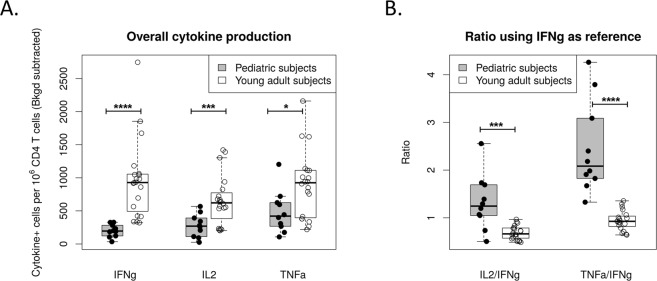
Overall cytokine production by influenza-specific CD4 T-cells is biased away from IFNγ in pediatric subjects. Following PBMC stimulation and staining, the frequency of activated IFNγ, IL-2 and TNFα producing cells per million CD4 T-cells was determined for each subject and stimulation condition. Cells producing each cytokine were then summed across stimulation conditions to quantify the total frequency of influenza-specific CD4 T cells for each cytokine examined. Data are presented as boxplots, with each point representing a given subject. In (**B**) these data were normalized by dividing by the frequency of IFNγ+ producing cells to better illustrate the differences in CD4 T cell cytokine production between young adults and children. Statistical analysis was performed by two-group comparison testing with the Wilcoxon rank-sum test after data preprocessing. P-values were adjusted for multiple comparisons using the Benjamini-Hochberg procedure.

**Figure 4:**
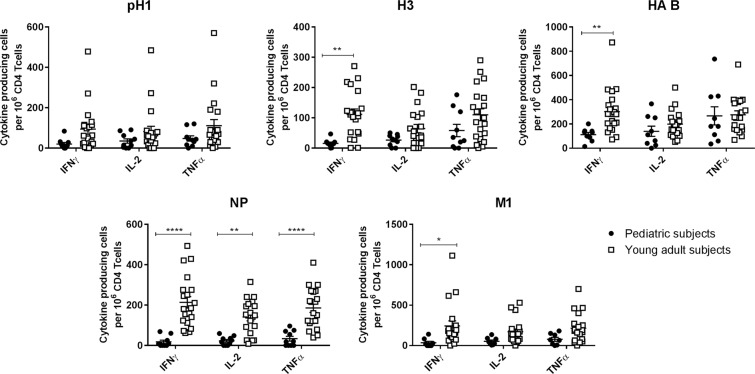
Pediatric subjects have a higher frequency of influenza-specific cells producing TNFα compared to IFNγ for all influenza proteins examined, with a global lack of NP-specific cells. Cells were stimulated and stained as described previously and then gated as in Fig. 1 to determine the frequency of activated, cytokine producing CD4 T cells for each stimulation condition. The frequency of influenza specific, cytokine producing CD4 T-cells was then graphed by individual stimulation condition. Statistical testing was completed using a 2-way ANOVA followed by *post hoc* comparisons with Sidak multiple comparison adjustment to assess for statistical significance between data obtained from children (solid circle) and young adults (open square).

Next, overall production of IFNγ, IL-2 and TNFα in pediatric compared to young adult subjects was examined by summing the frequency of cells producing each cytokine across the stimulation conditions examined (Fig. 3A). These data demonstrate that influenza-specific CD4 T cell responses are less Th1 biased in IIV vaccinated children compared to young adults, with higher frequencies of cells producing IL-2 or TNFα compared to IFNγ. This difference was highlighted by calculating cytokine production in each of the populations as a ratio normalized to the frequency of IFNγ+ CD69+ cells (Fig. 3B). When calculated in this manner, the ratio of cells producing IL-2 or TNFα compared to IFNγ was higher in pediatric compared to young adult subjects (IL-2/IFNγ p = 0.001; TNFα/IFNγ p < 0.0001). When the frequency of CD4 T cells producing IFNγ, IL-2, or TNFα was individually quantified for each of the proteins (Fig. 4), the pediatric population had a higher frequency of influenza-specific CD4 T cells producing TNFα compared to IFNγ across all conditions examined. Consistent with our data examining cellular specificity using IFNγ alone, the overall frequency of cells specific for the internal virion proteins (NP and M1) remained low for all cytokines quantified. Responses to the pH1, H3, and HA-B proteins, which are abundant in IIV, were more similar between the pediatric and young adult populations, especially when CD4 T cell frequency was quantified using IL-2 or TNFα as opposed to IFNγ.

**Figure 5:**
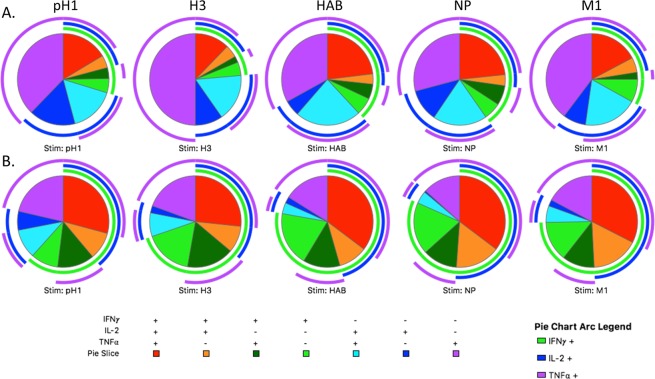
Cytokine production by CD4 T cells isolated from pediatric subjects is characterized by overall less IFNγ production, with the differences in cytokine elaboration between children and adults largely consistent across protein specificities. Cell were stimulated and stained as previously described, with gating as shown in Fig. 1. Combination gates were then created using FlowJo v10 software (TreeStar) to highlight single, double, and triple cytokine producing cells. Following data preprocessing, arced pie graphs were constructed using SPICE software (NIH), with the arcs demonstrating all cells producing a given cytokine (IFNγ: green, IL-2: blue, TNFα: purple) and pie slices depicting the percentage of cells with a given cytokine expression pattern as indicated in the Figure. Data from pediatric subjects are shown in Panel A, while young adult data are shown in Panel B.

**Figure 6:**
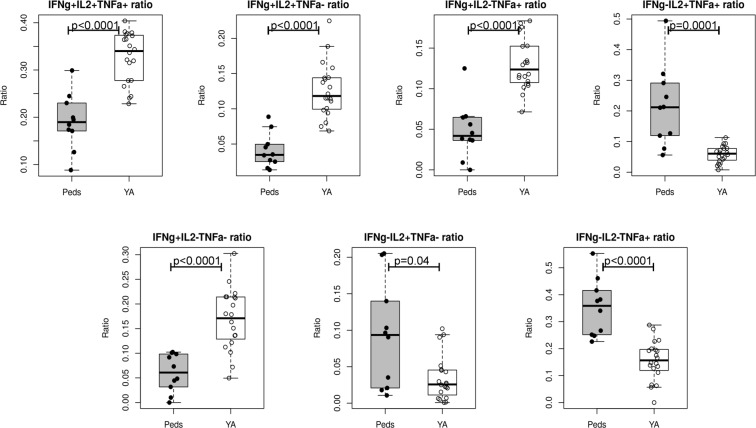
When summed across specificities, CD4 T cells from pediatric subjects are less likely to produce combinations of cytokines that include IFNγ and are more likely to produce TNFα or IL-2 alone or together. To better illustrate differences in cytokine production between the pediatric and young adult cohorts, subject-level data across each stimulation condition was summed for each cytokine combination and then was divided by the total number of cytokine-producing cells across all conditions. Data are presented as box plots with each point representing a given subject. Statistical testing was performed using the Wilcoxon rank sum test with the Benjamini-Hochberg procedure to correct for multiple comparisons.

Finally, as data suggests that polyfunctional CD4 T cells may be better able to promote pathogen clearance^24–26^, we used combination gating to determine the proportion of cells producing multiple cytokines simultaneously. This data is presented by stimulation condition for pediatric subjects in Fig. 5A and for young adult subjects in Fig. 5B. In addition, to facilitate statistical comparison between the groups, influenza-specific CD4 T cells elaborating each cytokine combination were summed across stimulation conditions and presented as a ratio of cells producing each cytokine combination compared to the total CD4 T cell frequency. This ratio is then presented as a box plot, with statistical testing performed between groups using the Wilcoxon rank sum test with P-values adjusted for multiple comparisons via the Benjamini Hochberg correction (Fig. 6). Results are presented with reference to the pie graphs in Fig. 5, with statistics derived using the summed overall data as presented in Fig. 6. When examined in this manner, the relatively low frequency of CD4 T cells producing IFNγ was again demonstrated, with a much smaller arc for IFNγ (green) in the pediatric (Fig. 5A) as compared to the adult (Fig. 5B) population. Interestingly, there was a relative dearth of polyfunctional cells overall detectable in pediatric subjects, with a lower proportion of cells able to produce IFNγ/IL-2/TNFα simultaneously (red pie; p < 0.0001) as well as a decreased percentage of cells producing either IFNγ/IL-2 (orange pie; p < 0.0001) or IFNγ/TNFα (dark green pie; p < 0.0001). However, this was likely the result of less CD4 T cell elaboration of IFNγ rather than a global deficit in the ability of CD4 T cells to produce multiple cytokines, as there were a greater proportion of cells producing the combination of IL-2 and TNFα in pediatric subjects (light blue pie; p = 0.0001). Pediatric subjects also had an increase in the percentage of cells producing TNFα alone (purple pie; p < 0.0001) and IL-2 alone (dark blue pie; p = 0.04), again reinforcing the presence of a less Th1-biased influenza-specific CD4 T cell response in children.

## Discussion

In this manuscript we detailed differences in CD4 T cell specificity and function between a pediatric population with limited influenza exposures compared to young adults who likely have had multiple previous encounters with influenza virus. We found that children had an overall lower frequency of influenza-specific CD4 T cells, most markedly to the internal virion protein NP. In addition, younger children had a less Th1-biased CD4 T cell response in comparison to the young adult cohort, with fewer polyfunctional cells and a reduced ability to produce IFNγ. These findings provide important insight into the evolution of the influenza-specific CD4 T cell response from childhood into adulthood.

All children in our study had previously been vaccinated with IIV but had no history of previous natural influenza infection or LAIV administration, suggesting that early childhood IIV immunization is able to prime CD4 T cell reactivity but that the memory established remains more undifferentiated and mainly directed against the HA proteins predominant within IIV^27,28^. While it is well established that IIV primes B cells and is able to establish an antibody response in young children^29,30^, the priming of cellular immunity in children post-IIV administration has been more controversial. He *et al*. demonstrated significant increases in IFNγ producing influenza-specific CD4 T cells in young children between 6 months to 4 years of age following vaccination, with a positive correlation between increasing age and baseline influenza-specific CD4 T cell reactivity^31^. Post-vaccination boosting of influenza-specific CD4 T cells was also reported by Reber *et al*. in a study of older children^32^. However, other groups have reported little priming of CD4 T cells following prime-boost administration of IIV in childhood^33,34^. Given the multiple functions of CD4 T cells in the anti-influenza immune response^13,35,36^ and the widespread use of IIV in infants in many countries, including the U.S.^37^, a better understanding of how early CD4 T cell immunity is established post-vaccination is critical.

The shift in CD4 T cell specificity and functionality between childhood and adulthood, marked by a gain in reactivity to internal virion proteins and a shift to greater production of IFNγ by influenza-specific CD4 T cells, suggests evolution of anti-influenza reactivity over time through repeated antigenic exposure. It is likely that the more robust inflammatory signaling associated with infection results in the development of a progressively more Th1 biased CD4 T cell response^38,39^. Similarly, CD4 T cells specific for epitopes within the more conserved internal virion proteins may gradually accumulate, especially after a naïve CD4 T cell response is initially primed by the high antigenic load produced by actively replicating virus^40^. Further research will be necessary to determine whether these changes in specificity and functionality are incited by a single infection or accrue gradually over the course of childhood following multiple independent exposures.

One unexpected finding in this study was the early establishment of influenza B HA immunodominance in the pediatric population. Our previous work has established that the HA-B protein is immunodominant among adult healthy donors^22^ despite a similar abundance of HA protein within influenza A and influenza B virions^41^. That this immunodominance pattern is established very early in childhood could be in part due to growing use of quadrivalent vaccines that contain twice the amount of the influenza B HA protein. Additional studies to evaluate whether this immunodominance pattern is preserved in young children following administration of a trivalent versus a quadrivalent vaccine could help to clarify the importance of antigenic dose in the early immunodominance of the HA-B protein demonstrated here.

Although we only quantified CD4 T cell elaboration of IFNγ, IL-2, and TNFα in this study, the pattern of cytokine expression demonstrated here may indicate an overall less differentiated CD4 T cell response in children. This, together with the dearth of memory CD4 T cells specific for epitopes within the more conserved internal virion proteins, could contribute to the increased morbidity seen in young children upon infection with a seasonal influenza virus. While consideration could be given to the use of vaccine adjuvants in order to more robustly prime a Th1 biased anti-influenza CD4 T cell response of broad specificity in children, how to utilize such immune activators to establish lasting immunologic memory against multiple influenza strains upon vaccination remains uncertain. Further, it is possible that it is precisely the lack of preexisting cell-mediated immunity in children that allows for the establishment of long-lasting B cell mediated immunity upon early influenza infection, as the greater antigen abundance and inflammatory signaling could lead to a more robust germinal center reaction^42^. The data presented here thus raise a multitude of questions regarding the mechanisms responsible for and consequences of changes in influenza-specific immunity between children and young adults. Studies to address these knowledge gaps are currently ongoing and will be critical to the development of next generation influenza vaccines able to provide durable protection against multiple influenza strains in populations of diverse age and exposure history.
